# Synthesis and characterization of a ZnGa_2_O_4_:Cr^3+^-based aerogel†

**DOI:** 10.1039/c9ra08303k

**Published:** 2019-10-22

**Authors:** Ting Zhang, Ai Du, Chuanxiang Chen, Xiujie Ji, Bin Zhou, Jun Shen, Zhihua Zhang

**Affiliations:** Shanghai Key Laboratory of Special Artificial Microstructure Materials and Technology, School of Physics Science and Engineering, Tongji University Shanghai 200092 China duai@tongji.edu.cn +86 21 6598 6071

## Abstract

A ZnGa_2_O_4_:Cr^3+^-based aerogel was prepared by using polyacrylic acid (PAA) as a dispersant and propylene oxide (PO) as a crosslinking agent *via* CO_2_ supercritical drying. The results of BET and SEM show that there is a certain degree of macroporosity (*d* > 50 nm) in the aerogel. It has a dendritic structure and the interior is relatively loose. EDS mapping illustrates that the elements Zn, Ga, and Cr are evenly distributed in the aerogel. In addition, the diffuse reflectance spectra and the emission spectrum of samples with different calcination temperatures were also characterized. Both demonstrated, when the calcination temperature is greater than 600 °C, that the sample crystallizes and has a significant emission, which is consistent with the XRD and TG-DSG results. Finally, the ZnGa_2_O_4_:Cr^3+^-based aerogel also exhibits excellent long afterglow performance and high photocatalytic performance with 80.1% methylene blue (MB) degradation at 20 min.

## Abstract 2

A ZnGa_2_O_4_:Cr^3+^-based aerogel was prepared by using polyacrylic acid (PAA) as a dispersant and propylene oxide (PO) as a crosslinking agent *via* CO_2_ supercritical drying.

## Graphical abstract

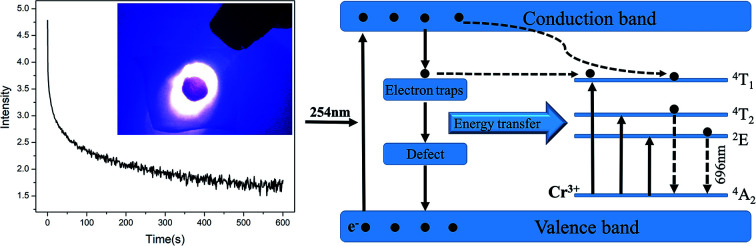

## 1. Introduction

As a self-activating new luminescent material, ZnGa_2_O_4_ has received extensive attention from researchers. It has excellent chemical stability and color purity.^1–4^ Therefore, this attractive oxide phosphor material is widely used for low-voltage cathodoluminescent and vacuum fluorescent displays, liquid crystal displays (LCD), field emission displays and electroluminescent devices.^5–10^ ZnGa_2_O_4_ is an AB_2_O_4_ spinel-type crystal structure.^4^ In terms of luminescence properties, the optical bandgap is about 4.4 eV. In the ZnGa_2_O_4_ single crystal, there are many defects, and some intrinsic defects are generated during the calcination process. In addition, due to the small difference in ionic radius, they will directly enter each other's crystal lattice. In ZnGa_2_O_4_, about 3% of antisite defects exist, which makes it have excellent long afterglow.^11,12^

As a good luminescent material matrix, it shows various emission colors when doped with different transition metal ions or rare earth ions, such as Cr^3+^, Mn^2+^, Eu^3+^ or Ce^3+^.^2,3,13–15^ The near-infrared luminescence of Cr^3+^ was reported as early as 1988, but the Cr^3+^ doped with ZnGa_2_O_4_ near-infrared luminescent material was rarely reported. The first report on near infrared-persistent luminescence in Cr^3+^ doped ZnGa_2_O_4_ by Bessière *et al.* in 2011.^16^ ZnGa_2_O_4_:Cr^3+^ is a bright red emitting long-lasting phosphor when ultraviolet excited with the emission range of 650–750 nm.

The reported ZnGa_2_O_4_:Cr^3+^ is mostly synthesized by high temperature solid state method. This method produces large particles, irregular morphology and sinter clustering. The researchers focused on finding a way to synthesize ZnGa_2_O_4_:Cr^3+^ at low temperatures and even in solution. Under this condition, it is beneficial to adjust the shape of the material and control its size. The sol–gel method gives an excellent control over the stoichiometry, introducing dopant easily and allowing lower processing temperature compared to other methods.^13^ The citrate sol–gel technique is the most common. But its only can obtained nanopowders instead of monolith. And the sample density is larger, the pores are less and the pore size is small.

A new sol–gel method for preparation of ZnGa_2_O_4_:Cr^3+^ long afterglow materials. In this paper, PAA and PO are used as a dispersant and a crosslinking agent to prepare a ZnGa_2_O_4_:Cr^3+^-based (with ZnO as the skeleton) aerogel. ZnGa_2_O_4_:Cr^3+^ has potential application value in bioprobe technology due to its excellent near-infrared long afterglow luminescence. The ZnGa_2_O_4_:Cr^3+^-based aerogel prepared by this method has more macropores, can be used as a drug carrier because of its sustained release effect. Also, monolith ZnGa_2_O_4_:Cr^3+^-based aerogel have a significant role in the field of hypervelocity particle capture. When space debris or comet particles impact this aerogel, as the depth of penetration increases, the aerogel heated temperature gradually decreases. Thus creating a temperature gradient on the track. Therefore, according to the difference of luminous intensity, the heating temperature at each position of the track can be derived. This is of great significance for solving the thermal interaction between hypervelocity particles and aerogels.

## 2. Experimental and characterizations

### 2.1 Materials

Zn(NO_3_)_2_·6H_2_O, Ga(NO_3_)_3_·*x*H_2_O, Gr(NO_3_)_3_·9H_2_O, polyacrylic acid (PAA, (C_3_H_4_O_2_)_*n*_), propylene oxide (C_3_H_6_O, ≥99.5%), ethanol (≥99.9%) were all purchased from Sinopharm Chemical Reagent Co., Ltd, Shanghai, China.

### 2.2 Preparation of ZnGa_2_O_4_:Cr^3+^-based aerogels

The ZnGa_2_O_4_:Cr^3+^-based gel was synthesized by sol–gel method using PAA as dispersant and PO as crosslinking agent. The Zn(NO_3_)_2_·6H_2_O, Ga(NO_3_)_3_·*x*H_2_O and Gr(NO_3_)_3_·9H_2_O were dissolved in ethanol at a molar ratio of 2 : 1 : 0.07. After 15 min of stirring, the solution was light blue, 2 ml of deionized water and 2.31 ml of PAA were added to the solution. After stirring for another half an hour, 5 ml of PO was added. Finally, the sol is placed at room temperature to gel. The gel was aged under airtight conditions for four days at room temperature and then soaked in ethanol for one week. Replaced with solvent ethanol three times, then carried out CO_2_ supercritical fluid drying. At last, ZnGa_2_O_4_:Cr^3+^-based aerogel was obtained. The dried sample was calcined in a muffle furnace at 500 °C, 600 °C, 700 °C, 800 °C for 2 h, respectively. The preparation process of the ZnGa_2_O_4_:Cr^3+^ aerogel (reference sample) with the atomic ratio of Zn : Ga = 1 : 2 is the same as above.

### 2.3 Photocatalytic experiment

Take 80 ml of methylene blue (MB) solution with a concentration of 20 mg L^−1^ in a culture dish, and then add 20 mg of aerogel powder samples. Place in the dark room for forty minutes to exclude the influence of adsorption on photocatalytic. Then, it was exposed to ultraviolet (UV) light for 100 min, and the concentration of methylene blue in each sample was measured by spectrophotometer every 20 minutes. We have made a calibration curve for methylene blue. The relationship between absorbance and its concentration is: *A* = 0.175*c* + 0.016. The photocatalytic experiment of the reference sample is the same as above.

### 2.4 Characterizations

The microstructure and morphology of the ZnGa_2_O_4_:Cr^3+^-based aerogel was characterized by a scanning electron microscope (SEM, Philips-XL30FEG, Thermo Fisher Scientific Inc., Hillsboro, OR, USA). The Brunner–Emmett–Teller (BET, AUTOSORB-1-MP, Quantachrome, Boynton Beach, FL, USA) was used to measure the pore size distribution and specific surface area of the aerogels. The samples with different calcination temperatures were also characterized by X-ray diffraction (XRD, X'Pert-Pro MPD, Holland Panalytical). The infrared absorption spectroscopy of aerogel after calcined was measured by Fourier infrared spectrometer (FTIR, Tensor 27, Bruker, Germany). A synchronous thermal analyzer (TG, STA449C, Netzsch, Germany) was used for recording TG-DSC curves of the aerogel. Photoluminescence (PL) spectra were determined by a Hitachi F-4500 spectrofluorometer. The diffuse reflectance spectra were measured by ultraviolet-visible-infrared (UV-Vis-IR) spectrophotometer (JASCO V-570, JASCO, Kyoto, Japan). (It is important to emphasize, the wavelength of the diffuse reflection detector is recorded according to the incident light wavelength.)

## 3. Results and discussion

**Fig. 1:**
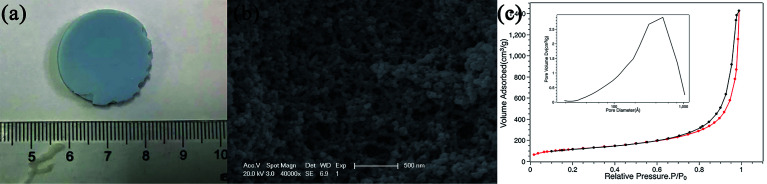
(a) The appearance, (b) SEM picture and (c) the N_2_ adsorption–desorption isotherms of ZnGa_2_O_4_:Cr^3+^-based aerogel, inset is the pore size distribution.

**Fig. 2:**
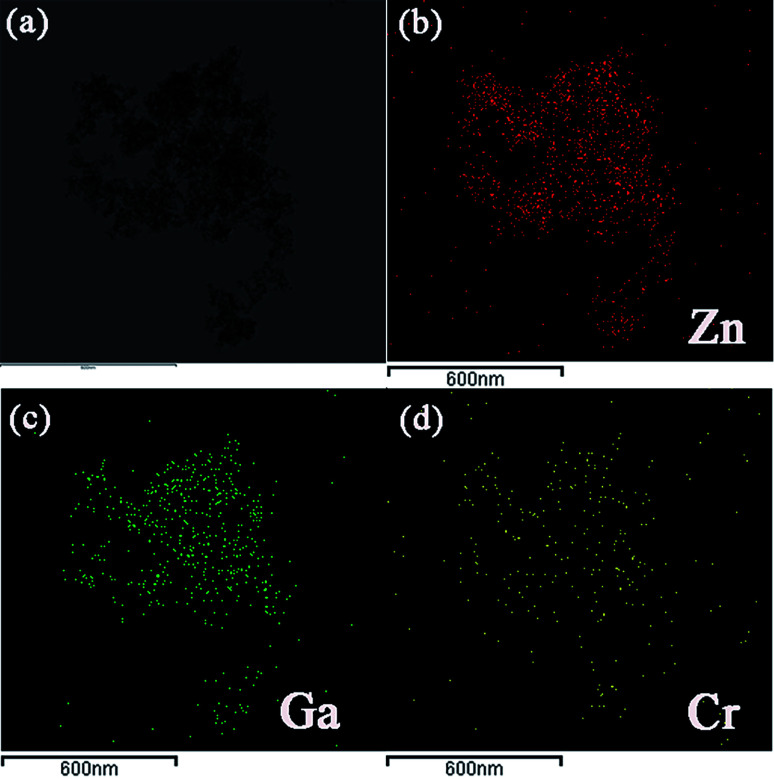
The (a) TEM picture of ZnGa_2_O_4_:Cr^3+^-based aerogel and EDS mapping images of (b) Zn, (c) Ga and (d) Cr element.

Fig. 1(a) shows the appearance of ZnGa_2_O_4_:Cr^3+^-based aerogel, which has good formability with a density of about 215 mg cm^−3^. Fig. 1(b) shows the SEM image. It can be seen that there are large holes, showing a dendritic structure and the interior is relatively loose. N_2_ adsorption–desorption isotherm and the pore size distribution of the aerogel are shown in Fig. 1(c). We can see that the pore size is widely distributed from big mesopores to macropores with an average value of 14.6 nm. And the specific surface area is 420.3 m^2^ g^−1^. The isotherm of the ZnGa_2_O_4_:Cr^3+^-based aerogel is type-IV, which has distinct capillary condensation and evaporation steps characteristic of mesoporous materials (*d* = 2–50 nm). There is no platform at high relative pressure, indicating a certain degree of macroporosity (*d* > 50 nm).^17^ It can also be seen from the pore size distribution image that the peak value is between 50–70 nm. The hysteresis loop shape of the aerogel is type-H1, which is characterized by parallel and nearly vertical branches, indicating the presence of a cylindrical pore with narrow pore size distribution or a large number of spherical holes continuously stacked.^18^ Fig. S1(a) and (b)† show that the reference sample has poor formability compared with ZnGa_2_O_4_:Cr^3+^-based aerogel. And a larger pore size in reference sample as illustrated in Fig. S1(c).†Fig. 2 is the (a) TEM picture of ZnGa_2_O_4_:Cr^3+^-based aerogel and EDS mapping images of (b) Zn, (c) Ga and (d) Cr element. The atomic percentages of the elements Zn, Ga, and Cr are 63.11%, 28.71%, 8.18%, respectively, and evenly distributed in the aerogel. The amounts of the Zn and Ga elements are basically the same as those mentioned in the experimental section.

**Fig. 3:**
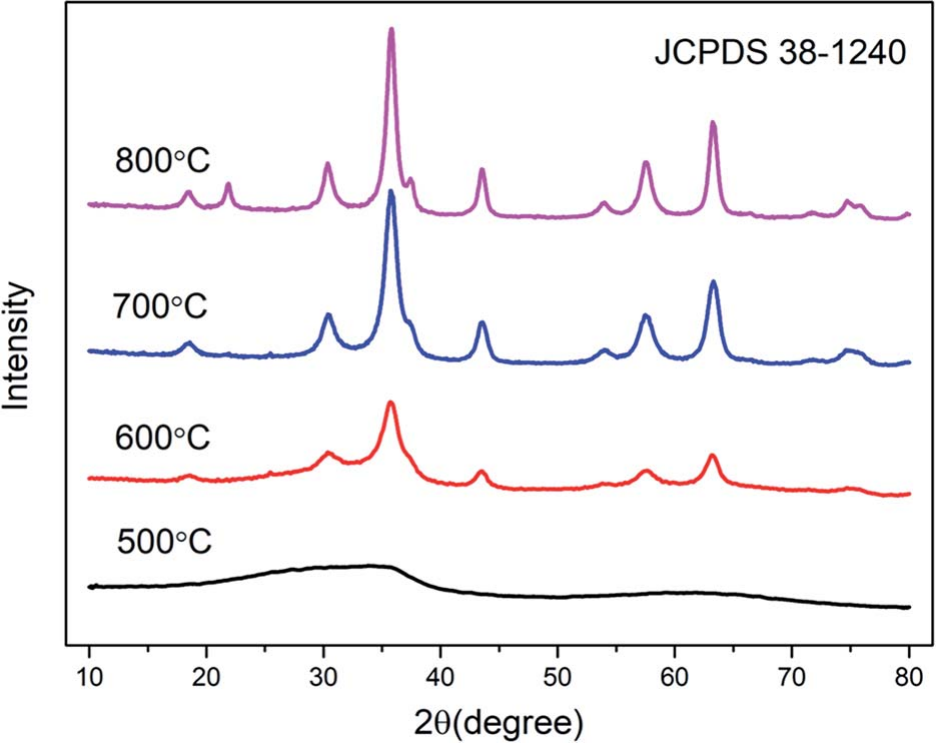
XRD patterns of ZnGa_2_O_4_:Cr^3+^-based aerogel as a function of calcined temperature.

Fig. 3 shows the XRD patterns of the samples with various calcined temperatures. It can be seen that the aerogel exhibits nanocrystalline properties after calcination at least 600 °C, and the sample calcined at 500 °C is amorphous. The peaks of the curves in the Fig. 3 correspond to No. 30-1240 in the JCPDS document, indicating that the sample is a cubic crystal spinel structure. The sample has crystallized at 600 °C and a broad diffraction peak appears. As the calcination temperature increases, the diffraction peaks gradually become stronger and sharper. We attribute it to an increase of crystallinity. It can be seen from Fig. S2† that the crystallization temperature of the reference sample increased from 600 °C to 700 °C.

**Fig. 4:**
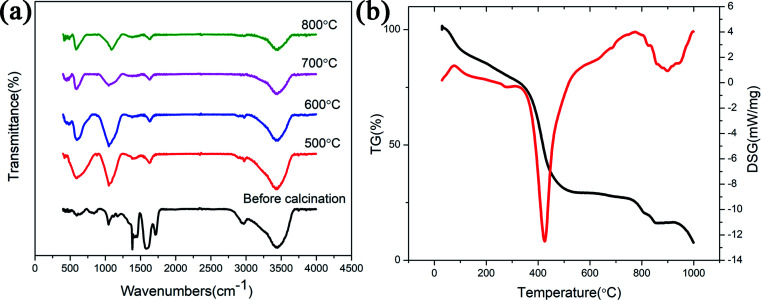
(a) FTIR spectra as a function of calcined temperature; (b) TG-DSG result of the ZnGa_2_O_4_:Cr^3+^-based aerogel before calcined.

FTIR spectra of the ZnGa_2_O_4_:Cr^3+^-based aerogel calcined at various temperatures are shown in Fig. 4(a). The broad band at 3430 cm^−1^ is due to the stretching vibration of H_2_O, indicating the existence of water absorbed in the samples. PAA commonly used as both a water absorbing agent and a dispersing agent. In this experiment, the aerogel prepared by using PAA as a dispersing agent is also easy to absorb water. The band at 1600 cm^−1^ represents the stretching vibrations of COO^−^ groups of PAA. It shows that the sample contains impurities and the organic matter is not completely decomposed. The band between 1000 cm^−1^ to 1300 cm^−1^ is due to the vibration of the NO^3−^ group. With the increasing of temperature, the above mentioned bands became weaker. Since the aerogel is based on ZnO, the Zn–O adsorption peak (589 cm^−1^) appears in the infrared spectrum of all samples. When the temperature reaches 600 °C, another adsorption peak appear at 420 cm^−1^ (Ga–O), which also indicating the formation of ZnGa_2_O_4_ nanocrystals. Unlike ZnGa_2_O_4_:Cr^3+^-based aerogel, there is no Zn–O (∼589 cm^−1^) adsorption peak (Fig. S3(a)†) in the uncalcined reference sample. When the temperature reached 700 °C, two adsorption peaks appeared at ∼586 cm^−1^ (Zn–O) and ∼420 cm^−1^ (Ga–O), indicating the formation of ZnGa_2_O_4_ nanocrystals. Fig. 4(b) shows TG-DSG results of the aerogel. The decrease in mass within 100 °C is attributed to the evaporation of adsorbed moisture. In the range of 100 °C to 360 °C, it can be seen slight weight loss due to decomposition of some organic groups. The strong exothermic peak at 410 °C accompanied weight loss is mainly due to the decomposition of PAA and some residual organic matter. The sample at 600–800 °C is in the endothermic process and the mass remains unchanged, indicating the formation of ZnGa_2_O_4_ nanocrystals. The crystallinity of the sample increases and the particles gradually increase during this process. However, an exothermic peak appeared at 900 °C should attribute to loss of ZnO during firing. The vapor pressure of ZnO is relatively high.^15^ But as shown in Fig. S3(b),† there is no excess ZnO in reference sample, so there is no exothermic peak at 900 °C.

**Fig. 5:**
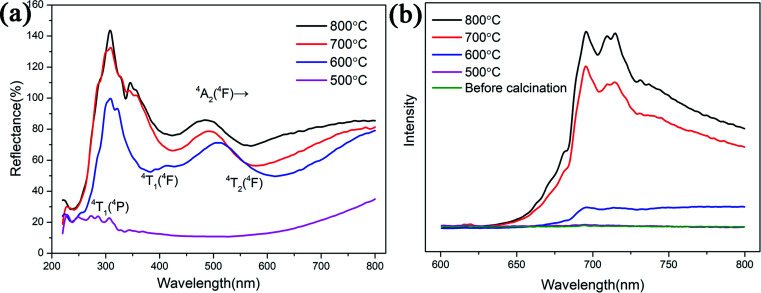
(a) The UV-visible diffuse reflectance spectra and (b) the PL emission spectrum of the ZnGa_2_O_4_:Cr^3+^-based aerogels with different calcination temperatures.

UV-visible diffuse reflectance spectra and the photoluminescence emission spectrum of the samples are shown in Fig. 5. The spectra of the samples both present two additional absorption bands at 550 nm and 430 nm which attributed to ^4^A_2_ (^4^F)–^4^T_2_ (^4^F) and ^4^A_2_ (^4^F)–^4^T_1_ (^4^F) transitions of Cr^3+^, respectively.^19,20^ The third peak is about 260 nm, we attribute this band to the spin-allowed ^4^A_2_ (^4^F)–^4^T_1_ (^4^P) absorption transition of Cr^3+^. Three excitation peaks attributed to three d–d absorption bands of Cr^3+^.^21^ From Fig. S4(a),† the UV-visible diffuse reflectance spectra of reference sample is the same as ZnGa_2_O_4_:Cr^3+^-based aerogel. It can be seen from the PL emission spectrum that the sample starts to glow after being calcined at 600 °C. This is consistent with the XRD results. Therefore, the UV-visible spectrum of the sample calcined at 500 °C cannot be excited. The higher the calcination temperature, the greater the luminous intensity. It can be seen from the Fig. 5(b) that the sample has a broad emission peak with a peak value of 696 nm. This typical emission peak is attributed to the ^2^E–^4^A_2_ transition of Cr^3+^ in the ZnGa_2_O_4_ luminescent matrix.^22^ The emission peak at 713 nm is ascribed to the short-range perturbations of the octahedral sites surrounding Cr^3+^. These perturbations are caused by crystal defects and can manifest in the form of separate bands.^23,24^ Fig. S4(b)† illustrates that the reference sample starts to glow after being calcined at 700 °C. Unlike ZnGa_2_O_4_:Cr^3+^-based aerogel, ZnGa_2_O_4_:Cr^3+^ aerogels not have the emission peaks at 696 nm and 713 nm, but a large span of light-emitting region at 700–950 nm. However, if aerogels are used in the field of hypervelocity particle capture, we prefer that materials emit red light instead of infrared. This makes it easy to observe the tracks with a microscope.

**Fig. 6:**
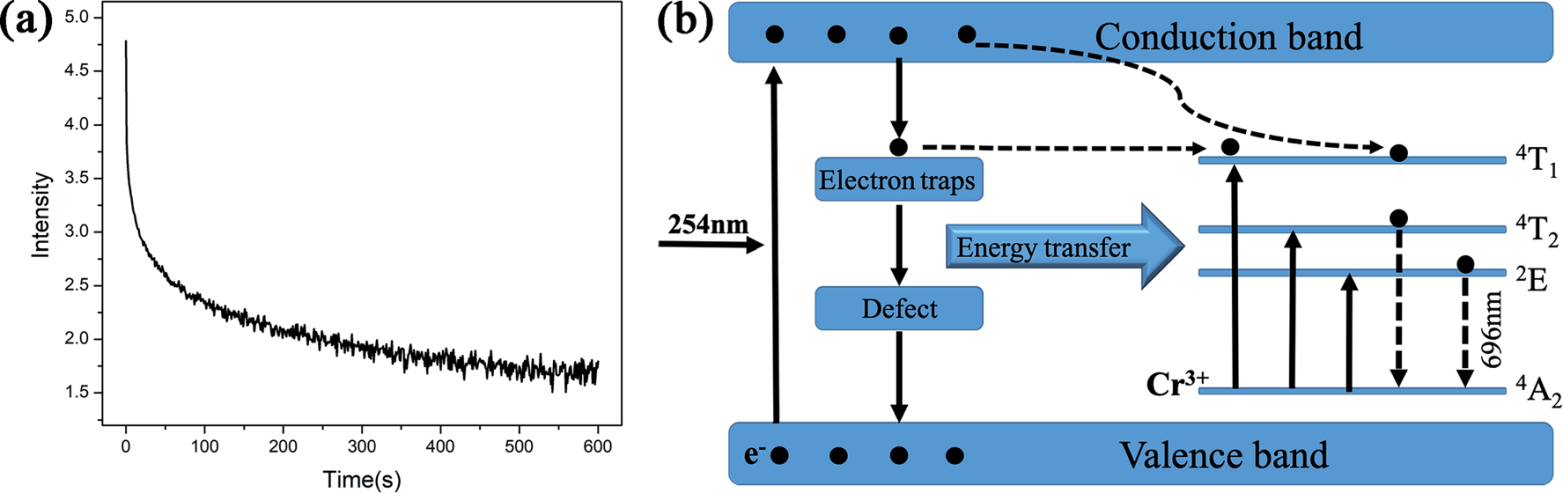
(a) Photoluminescence decay curve of the ZnGa_2_O_4_:Cr^3+^-based aerogel after 800 °C calcination, (b) the schematic diagram of the energy levels involved in the energy transfer mechanism between ZnGa_2_O_4_ and Cr^3+^ and the subsequent emission from Cr^3+^.

Since the luminescence intensity of the samples calcined at 800 °C is the highest, we have characterized its PL decay properties and as shown in Fig. 6(a). When the excitation light is removed, the fluorescence intensity drops to 1/*e* of the maximum fluorescence intensity *I*_0_ at the time of excitation, which is called the fluorescence lifetime *τ*. The bi-exponential represents two types of radiation channels, one is the emission between the conduction band and the valence band, and the other is the defect emission. The decay curves can be well fitted with a bi-exponential equation as given in the following expression:1
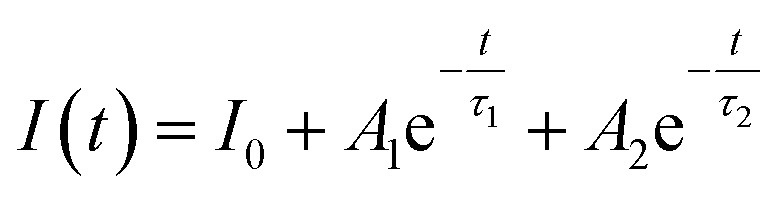
where *I* is the luminescence intensity at time *t*, *A*_1_ and *A*_2_ are constants, *τ*_1_ and *τ*_2_ are the decay times for the exponential components. The average lifetime (*τ*) can be determined using the following equation:

2 *τ* = (*A*_1_*τ*_1_^2^ + *A*_2_*τ*_2_^2^)/(*A*_1_*τ*_1_ + *A*_2_*τ*_2_)

The fitting data is shown in Table 1. The decay process of the long-lasting phosphorescence can be divided into two parts: the fast process and the slow process. And the decay time is mainly determined by the slow part. Fig. 6(b) illustrates the energy levels involved in the energy transfer mechanism between ZnGa_2_O_4_ and Cr^3+^ and the subsequent emission from Cr^3+^. The sample is excited by 254 nm UV light (high energy), and the incident photon is absorbed by the ZnGa_2_O_4_ (host), causing the electron to directly transition from the valence band to the conduction band. Electrons are transferred from the conduction band to the corresponding trap and captured due to thermal radiation and conduction band assist. The electron traps and hole traps are the energy states of storing excited electrons and holes, the energy can be stored for a period of time. However, after UV irradiation stopped, some electrons will break free from the shallow trap and transfer to the ^4^T_1_ energy level of Cr^3+^. The electrons trapped in the trap center transfer part of the energy to the Cr^3+^, causing the electrons of Cr^3+^ to excite from the ground state (^4^A_2_) to the three excited state levels of ^2^E, ^4^T_1_, and ^4^T_2_. The excited Cr^3+^ electrons return to the ground state by emitting photons of different energy.^13,25^ This produces a near-infrared long afterglow phenomenon which attribute to the sustained energy transfer of the host ZnGa_2_O_4_.^26^

**Table 1:** The fitting parameters and the calculated lifetimes from the PL decay curves of the ZnGa_2_O_4_:Cr^3+^-based aerogel after 800 °C calcination

*A*_1_	*τ*_1_ (s)	*A*_2_	*τ*_2_ (s)	*τ* (s)	*R* ^2^
7359.79	2.69	1430.79	36.06	26.81	0.98

**Fig. 7:**
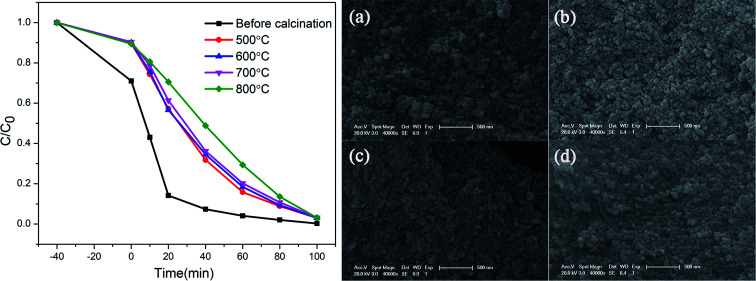
Photocatalytic effect of ZnGa_2_O_4_:Cr^3+^-based aerogels at different calcination temperatures; the SEM images of the samples after (a) 500 °C, (b) 600 °C, (c) 700 °C and (d) 800 °C calcination.

**Fig. 8:**
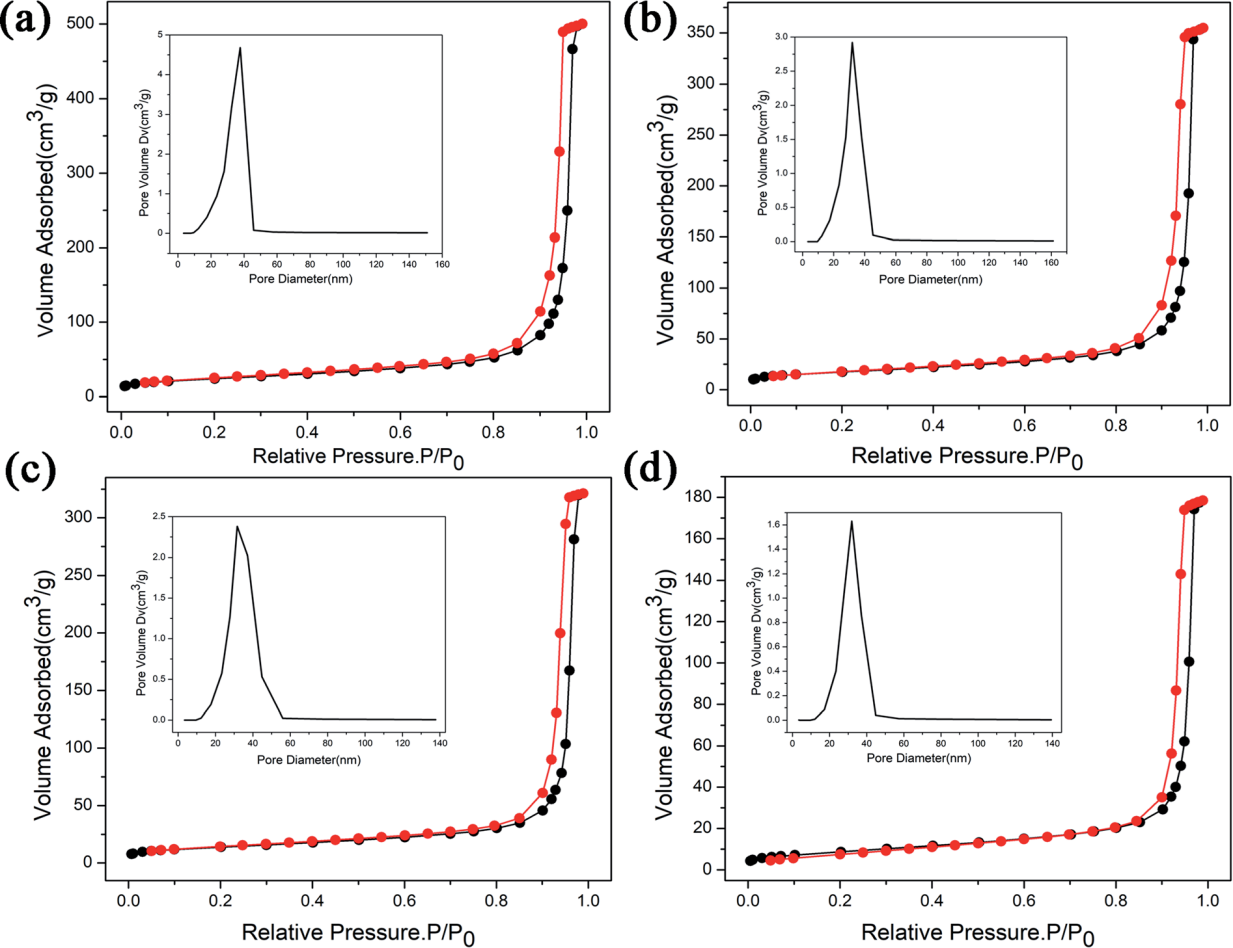
The N_2_ adsorption–desorption isotherms of the ZnGa_2_O_4_:Cr^3+^-based aerogels with different calcination temperatures, (a) 500 °C, (b) 600 °C, (c) 700 °C, (d) 800 °C, and inset is the pore size distribution of its.

The degradation of methylene blue (MB) dye was measured as a function of irradiation time of ZnGa_2_O_4_:Cr^3+^-based aerogels, and samples with different calcination temperatures was also tested for comparison. The sample without calcination exhibits high photocatalytic performance with 80.1% MB degradation at 20 min, much better than other materials. In addition, it can be seen that the adsorption effect of aerogel is also very good. After 40 minutes in the dark room, the adsorption rate reached 40%. However, the degradation rate of the calcined sample is slower and more uniform. The experimental results show that the heat treatment has a great influence on the photocatalytic and adsorption properties. As the calcination temperature gradually increases, the characteristic surface area of aerogels decrease and the particle volume increases, resulting in a decrease in catalytic activity. The morphology of the samples after calcined were characterized by SEM, as shown in Fig. 7. Obviously, the morphology changes from porous loose dendrites to dense spherical stacked structures. The pore structure almost disappears and the clusters are very tightly bound. This may be due to structural collapse of the sample during calcination. The SEM images are consistent with the conclusion obtained by photocatalytic. To support this conclusion, we characterized the N_2_ adsorption–desorption isotherms of calcined samples with different temperatures, as shown in Fig. 8. As the temperature increases, the specific surface area of the samples is 84.3 m^2^ g^−1^, 61.6 m^2^ g^−1^, 48.9 m^2^ g^−1^, 30.7 m^2^ g^−1^ (Table 2). Compared with Fig. 1(c), it is much reduced, once again proving this conclusion. In addition, photocatalytic and long-lasting luminescence are significantly associated. In the ZnGa_2_O_4_ host, a part of photo-generated electrons and holes recombine to release energy in the form of photoluminescence. And some of them can be trapped by lattice defects, impurities or Cr^3+^. The photo-generated electrons and holes are trapped to slow down the recombination process, which will enhance charge transfer and photocatalytic activity. On the other hand, after stopping the UV irradiation, some electrons and holes break free from the trap. Transferring part of the energy to Cr^3+^ and then emitting long-lasting light. It can be seen that the relationship between photocatalytic and long afterglow properties is mutually inhibited. As the calcination temperature increases, the luminescence intensity increases and the photocatalytic efficiency decreases.^27^

**Table 2:** The surface area and average pore size of the ZnGa_2_O_4_:Cr^3+^-based aerogels with different calcination temperatures

	Before calcined	500 °C	600 °C	700 °C	800 °C
Surface area (m^2^ g^−1^)	420.3	84.3	61.6	48.9	30.7
Average pore size (nm)	14.6	36.7	35.7	40.7	36.0

## 4. Conclusions

In this work, we have found a novel method to synthesize ZnGa_2_O_4_:Cr^3+^-based aerogel *via* CO_2_ supercritical drying. The results of BET and SEM show that there is a certain degree of macroporosity (*d* > 50 nm) in the aerogel. It has a dendritic structure and the interior is relatively loose. EDS mapping illustrates that the elements Zn, Ga, and Cr evenly distributed in the aerogel. In addition, the XRD, FTIR results and the diffuse reflectance spectra demonstrated when the calcination temperature is greater than 600 °C, the sample will exhibit nanocrystalline properties and have a significant emission. The PL spectrum of the sample shows that as the calcination temperature increases, the luminescence intensity increases. Furthermore, this materials exhibit excellent long afterglow performance and high photocatalytic performance with 80.1% methylene blue (MB) degradation at 20 min. We believe, this aerogel has a significant role in the field of hypervelocity particle capture. This is of great significance for solving the thermal interaction between hypervelocity particles and aerogels.

## Conflicts of interest

There are no conflicts to declare.

## Supplementary Material

RA-009-C9RA08303K-s001

## References

[cit1] Zhang W., Zhang J., Li Y., Chen Z., Wang T. (2010). Appl. Surf. Sci..

[cit2] Duan X. L., Yuan D. R., Wang L. H., Yu F. P., Cheng X. F., Liu Z. Q., Yan S. S. (2006). J. Cryst. Growth.

[cit3] Duan X., Liu J., Wu Y., Yu F., Wang X. (2014). J. Lumin..

[cit4] Shi Q., Wang C., Zhang D., Li S., Zhang L., Wang W., Zhang J. (2012). Thin Solid Films.

[cit5] Itoh S., Toki H., Sato Y., Morimoto K., Kishino T. (1991). J. Electrochem. Soc..

[cit6] Minami T., Kuroi Y., Miyata T., Yamada H., Takata S. (1997). J. Lumin..

[cit7] Omata T., Ueda N., Ueda K., Kawazoea H. (1994). Appl. Phys. Lett..

[cit8] Hsieh I. J., Chu K. T., Vu C. F., Feng M. S. (1994). J. Appl. Phys..

[cit9] Sei T., Nomura Y., Tsuchiya T. (1997). J. Non-Cryst. Solids.

[cit10] Jeong I. K., Park H. L., Mho S. (1998). Solid State Commun..

[cit11] Avci N., Korthout K., Newton M. A., Smet P. F., Poelman D. (2012). Opt. Mater. Express.

[cit12] Ye F., Dong S., Tian Z., Yao S., Zhou Z., Wang S. (2013). Opt. Mater..

[cit13] Hussen M. K., Dejene F. B., Gonfa G. G. (2018). Appl. Phys. A: Mater. Sci. Process..

[cit14] Kim J. S., Lee S. G., Park H. L., Park J. Y., Han S. D. (2004). Mater. Lett..

[cit15] Yu M., Lin J., Zhou Y. H., Wang S. B. (2002). Mater. Lett..

[cit16] Bessière A., Jacquart S., Priolkar K., Lecointre A., Viana B., Gourier D. (2011). Opt. Express.

[cit17] Kruk M., Jaroniec M. (2001). Chem. Mater..

[cit18] Aghajamali M., Iqbal M., Purkait T. K., Hadidi L., Sinelnikov R., Veinot J. G. (2016). Chem. Mater..

[cit19] Sharma S. K., Bessière A., Basavaraju N., Priolkar K. R., Binet L., Viana B., Gourier D. (2014). J. Lumin..

[cit20] Zhuang Y., Ueda J., Tanabe S. (2013). Appl. Phys. Express.

[cit21] Tanabe Y., Sugano S. (1954). J. Phys. Soc. Jpn..

[cit22] Srivastava B. B., Kuang A., Mao Y. (2015). Chem. Commun..

[cit23] Menon S. G., Choudhari K. S., Shivashankar S. A., Santhosh C., Kulkarni S. D. (2017). J. Alloys Compd..

[cit24] Cha J. H., Choi H. W. (2011). Trans. Electr. Electron. Mater..

[cit25] Hussen M. K., Dejene F. B. (2018). J. Sol-Gel Sci. Technol..

[cit26] Li L., Wang Y., Huang H., Li H., Zhao H. (2016). Mod. Phys. Lett. B.

[cit27] Li L., Wang Y., Li H., Huang H., Zhao H. (2015). RSC Adv..

