# Supplementary material for: Synthesis and characterization of a ZnGa2O4:Cr3+-based aerogel
Source: RSC Adv. 2019 Oct 22;9(58):33883–9. doi: 10.1039/c9ra08303k (PMC9073670; doi:10.1039/c9ra08303k)
Supplement: RA-009-C9RA08303K-s001 [file RA-009-C9RA08303K-s001.pdf]

Electronic Supplementary Material (ESI) for RSC Advances.
This journal is © The Royal Society of Chemistry 2019

Supporting Information

Synthesis and Characterization of $ZnGa_2O_4:Cr^{3+}$-based Aerogel

Ting Zhang, [a] *Ai Du,* * [a] *Chuanxiang Chen,* [a], *Xiujie Ji,* [a] *Bin Zhou,* [a] *Jun Shen* [a] *and Zhihua Zhang* [a]

[a] Shanghai Key Laboratory of Special Artificial Microstructure Materials and Technology, School of Physics Science and Engineering, Tongji University, Shanghai 200092, China.

[*] Correspondence: duai@tongji.edu.cn ; Tel.: +86 21 6598 6071

1. Results and discussion of reference sample

Fig. S1(a) and Fig. S1(b) show the appearance and the SEM image of $ZnGa_2O_4:Cr^{3+}$ aerogel with the atomic ratio of Zn:Ga=1:2 (reference sample), respectively. N_2 adsorption-desorption isotherms and the pore size distribution of reference sample are shown in Fig. S1(c). We can see that the pore size of reference sample is widely distributed from big mesopores to macropores with an average value of 22.7 nm. And the specific surface area is 426.6 m^2/g. Compared with $ZnGa_2O_4:Cr^{3+}$-based aerogel, the reference sample has poor formability and larger pore size.

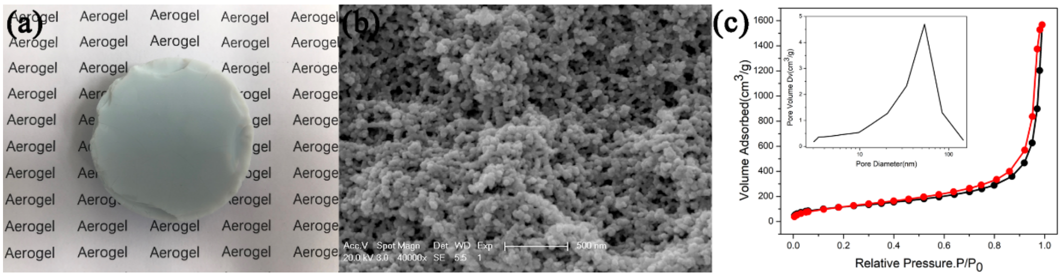

Fig. S1 (a) The appearance, (b) SEM picture and (c) the N_2 adsorption-desorption isotherms of the reference sample, inset is the pore size distribution.

Fig. S2 shows the XRD patterns of the reference sample with various calcined temperatures. It can be seen that the reference sample exhibits nanocrystalline properties after calcination at least 700 °C. The peaks of the curves in the Fig. S2 correspond to No. 30-1240 in the JCPDS document, indicating that the reference sample is a cubic crystal spinel structure.

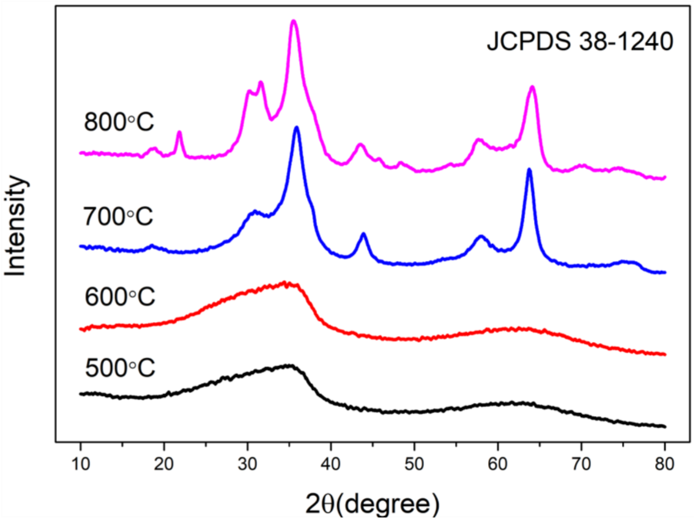

Fig. S2 XRD patterns of the reference sample as a function of calcined temperature.

FTIR spectra of the $ZnGa_2O_4$:Cr^{3+} aerogel calcined at various temperatures are shown in Fig. S3(a). Three distinct adsorption peaks at 3430, 1600 and 1100 cm^{-1} also appeared in the aerogel, representing H_2O, COO^- and NO^{3-} stretching vibration, respectively. Unlike $ZnGa_2O_4$:Cr^{3+}-based aerogel, there is no Zn-O (~589 cm^{-1}) adsorption peak in the uncalcined sample. When the temperature reaches 700 °C, two adsorption peaks appear at ~586 cm^{-1} (Zn-O) and ~420 cm^{-1} (Ga-O), indicating the formation of $ZnGa_2O_4$ nanocrystals. Fig. S3(b) shows TG-DSG result of the $ZnGa_2O_4$:Cr^{3+} aerogel. The decrease in mass within 100 °C is attributed to the evaporation of adsorbed moisture. In the range of 100 °C to 330 °C, it can be seen slight weight loss due to decomposition of some organic groups. The strong exothermic peak at 390 °C accompanied weight loss is mainly due to the decomposition of PAA and some residual organic matter. The reference sample is in the endothermic process at 600 °C ~ 1000 °C and the mass remains unchanged, indicating the formation of $ZnGa_2O_4$ nanocrystals, during which the crystallinity of the sample increases and the particles gradually increase. In the $ZnGa_2O_4$:Cr^{3+} aerogel, there is no excess ZnO, so there is no exothermic peak at 900 °C.

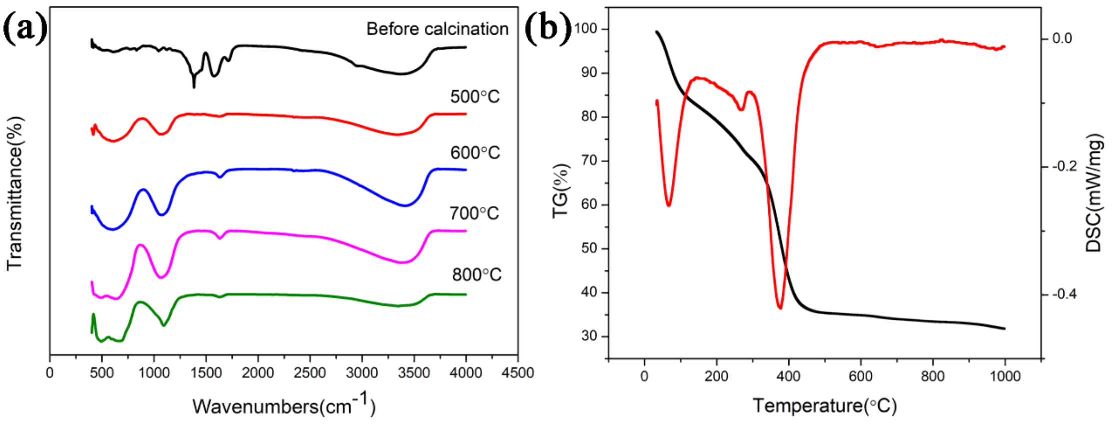

Fig. S3 (a) FTIR spectra as a function of calcined temperature; (b) TG and DSG results of the reference sample before calcined.

UV-visible diffuse reflectance spectra and the photoluminescence emission spectrum of the reference sample are shown in Fig. S4. Also, three excitation peaks attributed to three d-d absorption bands of Cr^{3+}. From the PL emission spectrum, it can be seen that the sample starts to glow after being calcined at 700 °C. Unlike $ZnGa_2O_4:Cr^{3+}$-based aerogel, the reference sample not have the emission peaks at 696 nm and 713 nm, but a large span of light-emitting region at 700 nm-950 nm.

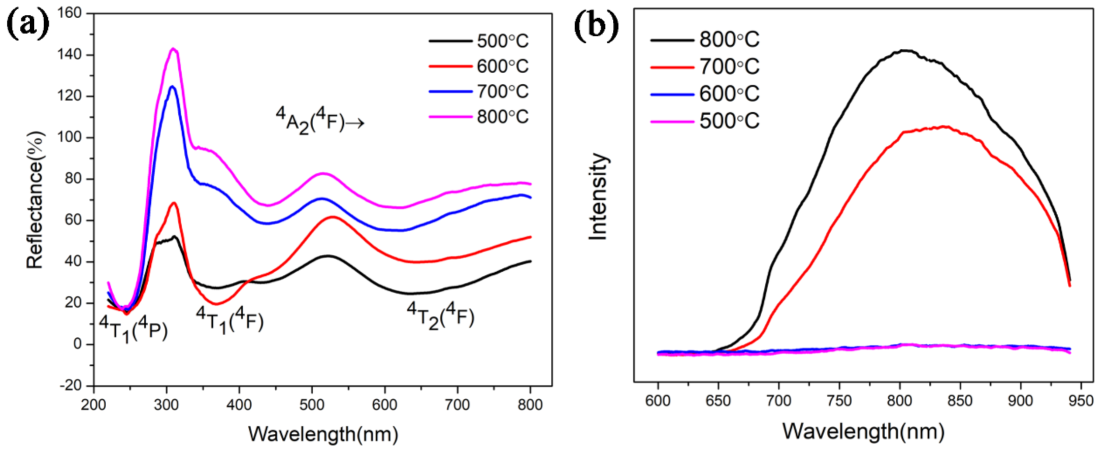

Fig. S4. (a) The UV-visible diffuse reflectance spectra and (b) the PL emission spectrum of the reference sample with different calcination temperatures.
